# Parental coping with uncertainties along the severe combined immunodeficiency journey

**DOI:** 10.1186/s13023-022-02554-9

**Published:** 2022-10-27

**Authors:** Oksana Kutsa, Sara M. Andrews, Erin Mallonee, Angela Gwaltney, Alissa Creamer, Paul K. J. Han, Melissa Raspa, Barbara B. Biesecker

**Affiliations:** 1 GenOmics, Bioinformatics, and Translational Research Center, RTI International, Research Triangle Park, NC USA; 2 Immune Deficiency Foundation, Towson, MD USA; 3 Behavioral Research Program, National Cancer Institute, Bethesda, MD USA

**Keywords:** Severe combined immunodeficiency, Coping with uncertainties, Adaptation, Rare genetic diseases

## Abstract

**Background:**

Severe combined immunodeficiency (SCID) is a group of rare genetic disorders that cause disruption in immune system functioning. Parents of children with SCID experience many uncertainties related to their child’s diagnosis, treatment, recovery, and quality of life. To fully understand parents’ experiences throughout their SCID journey, it is important to explore the stressors generated by such uncertainties and how parents cope with these stressors.

**Methods:**

We conducted 26 in-depth interviews with parents whose child was diagnosed with SCID or a SCID-like condition through newborn screening. The interviews explored uncertainties related to their child’s diagnosis and how parents coped with these uncertainties. Transcripts were generated from the interviews and analyzed using an inductive content analysis approach which included data immersion, generation and assignment of codes, and interpretation.

**Results:**

Parents used a variety of behavioral, cognitive, and affective coping strategies which evolved throughout their SCID journeys. Some parents reported coping by playing an active role in their child’s treatment, which included reaching out to other SCID parents or seeking second medical opinions. Other types of coping included establishing house hygiene rules, thinking positively about the child’s treatment progress, and relying on family members for help. These coping strategies were both deliberate and intuitive. Participants also described their struggles in coping with stressors related to their child’s health and survival. They reported difficulty in processing their emotions and experiencing denial and guilt related to their child’s diagnosis. Some parents adapted to ongoing uncertainties through such strategies as positive thinking, self-reflection, and relying on family and community. With successful adaptation, parents emphasized that they continue to use these strategies today.

**Conclusion:**

Our assessment revealed that parents of children diagnosed with SCID use a variety of behavioral, cognitive, and affective approaches to cope with SCID uncertainties. Although parents reported challenges in coping with SCID uncertainties, they also reported finding ways to overcome these stressors and establish patterns of effective coping. Findings from our study can serve as a guide for parents whose child was newly diagnosed with SCID and for providers such as social workers, genetic counselors, and psychologists.

**Supplementary Information:**

The online version contains supplementary material available at 10.1186/s13023-022-02554-9.

## Introduction

### Severe combined immunodeficiency syndrome: a source of parental uncertainties

Severe Combined Immunodeficiency (SCID) describes a group of rare genetic disorders characterized by extreme deficits in immune functioning, which leaves affected individuals highly susceptible to frequent, life-threatening infections [1, 2]. Prior to the development of hematopoietic stem cell transplantation (HSCT) as a treatment for SCID in 1968, the condition was universally fatal, often in infancy [3, 4]. Treatments, which also include experimental gene therapies for certain types of SCID, have been found to be most successful if delivered prior to the onset of infection [5]. SCID-related morbidity and mortality have significantly declined since the introduction of newborn screening for SCID in 2018 [5] because of earlier diagnosis leading to timely treatment. Despite improvements in SCID diagnoses and treatments, SCID remains a life-threatening condition, and parents of children diagnosed with SCID face significant psychological challenges related to their child’s condition [6].

To better understand the experiences of parents whose child was diagnosed with SCID, we previously reported on a mixed-methods study [6] that revealed parents’ experiences with SCID may be divided into five stages, collectively described as the SCID journey. The SCID journey encompasses parents’ experiences with receiving their child’s abnormal newborn screening results and receiving a diagnosis of SCID or a related condition, then proceeding to pre-treatment decision making, treatment, post-treatment, and adapting to a new life after their child returns home from the hospital. Table 1 depicts these stages of the SCID journey and the events that may occur at each stage. Notably, the SCID journey is unique to each parent and not necessarily a linear process. For example, parents may progress to the new normal stage and then return to the pre-treatment stage if the child’s initial treatment fails. Our study suggested that parents’ experience of the SCID journey includes perception of many uncertainties related to their child’s treatment, survival, and long-term health [6].

**Table 1:** SCID Journey Stages

SCID journey stage	Description
Diagnosis	Parents receive their child’s abnormal SCID screening results and follow up with their child’s provider. SCID diagnosis is confirmed, and parents are often referred to a specialist to discuss next steps for their child’s treatment
Pre-treatment	The child is admitted to the hospital or sent home and isolated. Treatment options, either HSCT, also known as bone marrow transplantation (BMT), or gene therapy are discussed/decided
Treatment	If BMT is selected as a treatment option, donor match is found. If required, chemotherapy is administered, followed by the selected treatment (BMT or gene therapy)
Post-treatment	Parents are waiting at or near hospital/treatment center to determine if treatment takes effect. This waiting period can take up to several months. The baby is discharged/sent home if treatment is effective. More treatment may be necessary
New normal	Parents and the child return home. The family tries to adapt to life after treatment. The child still requires continued follow-up and may require some medication. This stage can last from a few months to years

Although uncertainties are not uniformly appraised as stressors, uncertainties surrounding an illness that affects one’s loved one are commonly perceived as a threat [7]. In our prior research with parents of children with SCID, uncertainties related to their child’s condition were conveyed as a significant and ongoing source of stress throughout their SCID journey [6]. This is unsurprising given the trauma of an unexpected and life-threatening diagnosis in a newborn that is psychologically disruptive and results in parents feeling out of control [6]. Yet limited evidence has been reported on how parents cope with myriad uncertainties when their child is diagnosed with a condition like SCID in which treatment options are relatively new and the long-term post-treatment prognosis is unknown.

### Background: coping with uncertainty in illness

Evidence from the literature on coping with uncertainties suggests that threat appraisal is an initial step toward coping, as individuals strive (consciously and unconsciously) to restore a sense of well-being by managing the impact of the negative thoughts and feelings generated by the threat [7, 8]. Mishel developed the *Uncertainty* in Illness Theory (1988) that first proposed that uncertainty about illness may be perceived as a threat or an opportunity [9]. As a threat, it manifests as stress that is disruptive to the individual. To effectively adapt to the condition, coping with the condition and its stressors is essential. When illness uncertainty is perceived as an opportunity, it is proposed as adaptive to living with the condition [7] and has been shown to be associated with hope [10, 11].

Lazarus and Folkman’s Transactional Theory of Stress and Coping, the most widely used model to frame research on adaptation to a chronic health threat, expands on Mishel’s work in detailing how stressors are defined and appraisals of threat are assessed. Stressors result from an imbalance between the degree of threat and one’s perceived ability to address it [7]. When faced with a stressor, one assesses the potential threat (primary appraisal) and one’s ability to manage it (secondary appraisal). Secondary appraisal of the stressor informs the type of coping one subsequently employs. Problem-focused coping seeks to change the stressor or one’s relationship to it, while emotion-focused coping seeks to regulate emotional distress from managing the stressor [8]. Within these two categories there exist examples of what may be described as cognitive coping: that which seeks to change the way one perceives or thinks about a stressor to reduce its threat and perceive it as more manageable. Over time, effective coping facilitates adaptation to a stressor such as illness uncertainty. Adaptation is defined in this theory as attainment of emotional well-being and restored functional status when coping approaches are effective.

### The threat of a SCID diagnosis in a newborn

Studies of parents of children with cystic fibrosis, sickle cell anemia, fragile X syndrome, and “rare” undiagnosed conditions identify effective coping as a predictor of positive adaptation to their child’s condition [12–14]. Regardless of these encouraging results, and significant treatment advances, SCID remains a life-threatening condition resulting in long-term outcomes that are difficult to predict. SCID-related uncertainties can result in stressors that remain with parents for many years. There is little prior literature on how parents of children with SCID cope with the uncertainties that arise at different points in the journey. As such, we undertook a qualitative study that emerged from our prior work with SCID parents to better understand the many uncertainties emanating from their SCID journey and identify ways parents find to cope with them.

## Methods

### Participants

To be eligible for this study, parents had to be at least 18 years of age, speak English, reside in the United States, and have a child with SCID or a SCID-like condition identified through newborn screening. Only one parent of the same child was eligible to participate. To recruit parents, we partnered with a patient advocacy organization, SCID Angels for Life Foundation. After parents expressed initial interest in an announcement about the study in the foundation’s (closed) Facebook group, they were contacted to determine their eligibility and be provided details of the study. After a representative from the SCID Angels for Life Foundation confirmed eligibility, parents were formally invited to participate via email from the research team at RTI International. In total, 37 parents expressed interest in taking part in the study, and 7 did not meet the eligibility criteria.

Prior to participating in the interview, all participants provided informed consent to participate and completed a short sociodemographic questionnaire. In total, 26 parents whose children had different types of SCID or SCID-like conditions provided consent, completed the demographic questionnaire, and participated in the interviews (Table 2). X-linked SCID, with mutations in the IL2RG gene, was the most common form of SCID among the children whose parents participated in the study.

**Table 2:** Demographics

	N = 26 N (%)
Relationship to child	
Mother	21 (81%)
Father	5 (19%)
Child’s age mean (months)	4.5
Child’s type of SCID	
ADA	3 (12%)
Artemis	1 (4%)
Ataxia telangiectasia	1 (4%)
Complete DiGeorge	1 (4%)
DiGeorge syndrome	1 (4%)
IL2RG	10 (38%)
IL7R	1 (4%)
Nijmegen breakage syndrome	1 (4%)
RAG1	1 (4%)
RAG1/Omenn’s	1 (4%)
Unknown SCID	4 (15%)
Awaiting results	1 (15%)
Child’s journey stage	
Diagnosis	0
Pre-treatment	4 (15%)
New normal/pre-treatment	2 (8%)
Post-treatment	1 (4%)
New normal/post-treatment	4 (15%)
New normal	15 (58%)

### Data collection

In-depth interviews were conducted virtually between October and November 2020 via a video conferencing platform. The interviews were conducted by two researchers (SA and AG) with experience in qualitative research methodology. A semi-structured interview guide was used to primarily explore parental uncertainties related to their child’s diagnosis at each stage of the SCID journey. Parents were also asked how they coped with the uncertainties they described. Example questions are presented in Table 3 and the full guide is attached as part of Additional file 1. Interviews lasted 60 min on average, and the interviewers took extensive notes during the interviews using a structured template. All interviews were audio and video recorded and transcribed verbatim. Participants were offered $100 in appreciation for their time.

**Table 3:** Interview guide example questions

*Coping strategies*	
What, if anything, [has helped/is helping] you manage [OR cope with] this [source of uncertainty]? What (else), if anything, [would have been/would be] helpful for managing [source of uncertainty]?	If you could give advice to another SCID parent about managing [source of uncertainty they are talking about], what would you say?
*Changes in coping strategies*	
Have you noticed any differences in how you respond to [OR cope with] uncertainties in your journey with [Child’s name] now compared to when [he/she] was first diagnosed?	Do you feel that the ways you are managing uncertainty today are working better?

### Data analysis

Coding and analysis of all data were conducted in NVivo 12. We conducted an inductive content analysis, including immersion in the data, generation and assignment of codes that represented key aspects of the data (e.g., coping strategies), categorization of coping codes into overarching specific coping approaches, and interpretation [15, 16]. Using the preliminary findings obtained from interview notes and review of transcripts, the research team, consisting of coders (OK and EM), interviewers (SMA and AG), and senior researchers with expertise in newborn screening and rare diseases (MR), health behavior (BBB), and uncertainty (PKJH) developed or provided feedback on the initial list of reported coping mechanisms used throughout the child’s SCID journey. The coping codes were refined through discussion among the coders and the research team during double-coding of the first six transcripts. The coders reached consensus in code refinement by discussing the challenges in consistently applying the codes, proposing changes, and agreeing on any code modifications. If the coders were unable to reach consensus, the research team mediated any discussions related to code changes [17]. To establish interrater reliability (IRR), a total of eight (30%) of the transcripts were double-coded. After obtaining a Cohen’s kappa of 0.78 across the first eight transcripts, which signified substantial agreement between the coders [18], the coders began coding independently. Three additional transcripts were double-coded, at intervals throughout the independent coding of 15 transcripts, to ensure that coders maintained high levels of IRR throughout the coding process. The final codebook is attached as part of the Additional file 2 which includes a listing of the coping codes and examples.

After applying the coping codes to the transcripts, BBB, OK, and SMA reviewed the most frequently reported coping mechanisms at each stage of the SCID journey and categorized them by type of coping (Table 4). Coping strategies explored included behavioral, cognitive, and affective approaches. Behavioral coping strategies largely reflected parents’ efforts to change their actions to manage the stress generated by SCID-related uncertainties [19]. Cognitive coping, such as reframing, reflected parents’ efforts to change how they were thinking about SCID uncertainties to manage the cognitive dissonance that results [20]. Affective coping was executed to manage negative feelings generated by SCID-related uncertainties [21]. This type of categorization is complex because the coping mechanisms parents reported may represent a combination of behavioral, cognitive, and affective strategies. We selectively report whether a specific coping mechanism appeared most often as behavioral, cognitive, or affective, in terms of whether the mechanism resulted in changes in behavior, thought processes, or feelings.

**Table 4:** Summary of coping mechanisms by stage

Coping	Diagnosis	Pre-treatment	Treatment	Post-treatment	New normal
Behavioral	**Active seeking of SCID-related information** *Ex: Researching information about SCID, talking to other SCID parents*	**Playing an active role in treatment decisions** *Ex: Asking physicians questions, consulting with other families about treatment options* **Placing trust in the treatment team** *Ex: Following physician’s guidance and recommendations*	**Playing an active role in treatment decisions** *Ex: Tracking child’s medication, monitoring compliance with hospital hygienic protocols*	NA	**Identifying what is actionable and controllable** *Ex: Establishing house rules and hygiene protocols, taking the child to physical therapy*
Cognitive	NA	NA	NA	NA	**Focusing on what is actionable and controllable** *Ex: Thinking positively about child’s progress*
Affective	**Placing trust in the treatment team** *Ex: Relying on providers and their expertise, having active conversations with providers about treatment options*	**Placing trust in the treatment team** *Ex: Trusting physician’s expertise on treatment options*	NA	**Relying on help from others** *Ex: Allowing relatives to take care of children staying at home, working with care coordinators on questions about insurance or appointment scheduling*	NA

Most unexpected life-threatening events present as crises. As parents learn of the SCID diagnosis, they are generally overwhelmed by the unexpected life-threatening nature of the diagnosis. As such, it is difficult to appreciate the full extent of SCID-related uncertainties that they face. In a crisis, it is challenging to process information, reflect on its impact, and generate a plan forward. Some of what parents described as coping in the first days of learning about their child’s SCID diagnosis likely reflects their efforts to understand the degree of threat to their child’s life. Their initial recognition of the uncertainties they faced likely represented their responses to the threat of their child’s life. With time, those responses became conscious and deliberate recognition of uncertainties and ways forward that led to their initial efforts at coping described herein.

## Results

### Coping in the diagnosis stage

At the beginning of their SCID journey, parents experienced uncertainties about SCID and its causes, as most often the diagnosis was unexpected. After receiving an abnormal screening result for SCID, parents had many questions about the condition and its impact on their child’s health. Immediately following their child’s diagnosis, parents reported feeling confused and anxious. Some parents did not receive an official diagnosis of SCID after an abnormal screening result, whereas others received a diagnosis that was not SCID but a related immunodeficiency. Parents most often reported using behavioral and affective coping to manage these uncertainties they experienced upon learning their child’s diagnosis.

#### Behavioral coping

Parents reported *actively seeking out SCID-related information*, for example by researching information about SCID online, searching for other parents who had a child with SCID, and seeking clinical specialists who could provide second opinions on the diagnosis and information about potential treatment options. One parent described waiting for his child’s confirmatory testing results and using online research to learn more about SCID and other SCID-related conditions:

*They [doctors] got the blood test, and then they escorted us out the back so that he [the child] wouldn’t go through like a public area, and we were kind of concerned. They gave us a little bit of details about what they were looking for. They said that he [the child] had a very low T-cell count, but they wouldn’t say exactly what it was. So, my wife started doing some research online and kind of looking at different things and, you know, there’s only a few options of what it could be, it was either going to be SCID or DiGeorge syndrome.*

#### Affective coping

In the diagnostic stage of the SCID journey, when parents had questions about the genetic causes of SCID, whether there were treatment options available, and what their child’s prognosis was, they often relied on their medical providers in ways that facilitated affective coping. They *trusted the treatment team* to reliably care for their child. Parents reported feeling “calmer” after their medical providers explained the cause of SCID and reviewed next steps and availability of various treatment options. Trusting physicians’ expertise provided parents with some relief about the difficult decisions that they would have to make around their child’s diagnosis and treatment. Their expressed regard for the providers allowed parents to feel more at ease in the face of uncertainties about their child’s future. This is reflected by one parent who stated:

*When we [the child] were diagnosed, we were sent to [Hospital Name] in [City]. And they had a wonderful team there and very knowledgeable, you know, they were answering all our questions and help put our mind at ease. But it was so new to us that, well, like it is probably to most families*.

### Coping in the pre-treatment stage

After parents received a confirmed diagnosis for their child, their uncertainties shifted to center on treatment options. In some cases, parents were presented with two options: a bone marrow transplant that is a recognized treatment for SCID or an experimental gene therapy as part of a clinical trial. In addition to uncertainties about treatment options, parents faced decisions about treatment location and whether to undergo pre-treatment chemotherapy. Parents also relayed uncertainties about the cost of treatments, whether their health insurance would cover them, and how to care for other family members while keeping their newly diagnosed baby safe in isolation. Parents often felt overwhelmed and anxious during this stage. Parents also experienced sadness and depression. Overall, they reported use of both behavioral and affective coping to manage uncertainties in the pre-treatment stage.

#### Behavioral coping

Parents reported behavioral coping by *playing an active role in decisions* related to their child’s treatment. To make these decisions, parents actively engaged in considering the advantages and disadvantages of available treatment options, asking physicians questions, and eliciting the experiences of other families whose child has the same type of SCID. By taking an active part in their child’s pre-treatment and treatment decisions, parents felt more confident that they were selecting a treatment that was most appropriate for their child. One parent described managing uncertainty associated with pre-treatment decision-making as follows:

*So what we did was we made a kind of a pro/con chart, you know, we sat down and we wrote down, I wrote out all the different treatment options and, you know, what the positives and negatives are of each, which ones would need chemotherapy, which ones wouldn’t, and what possible side effects could happen and stuff like that. And that was a really, really important tool for us to help make the decisions.*

In some instances, parents had to actively make treatment decisions after the first round of treatment for their child was unsuccessful. One parent reported actively looking for other treatment options when her child’s first transplant did not work:

*And then when they found out when the first transplant didn’t work, I said, let’s look at [child’s brother as a donor], you know, to see if he’s a match. And they didn’t want to do that [due to concerns about sibling’s size and age]. They said, ‘We can’t do it. Let’s do the stem cell [referring to HSCT with cord blood that was 5 out of 6 match].’ So, I had to push, I had to ask questions and keep looking. And I saw a status up on the Facebook group, and that kind of directed me and sent me to the doctors that had the experience that said, ‘yes, [sibling] could be the donor.’ There’s a lot of success with cord blood. But since my [child] had a better alternative [the sibling], and [the doctor she found via the Facebook group] said it could be done…that’s where everything went successful.*

Parents also reported *putting trust in their treatment team* in ways that reflected behavioral coping. For example, parents often acted on doctors’ guidance, descriptions of available treatment, and recommendations for their child’s treatment despite uncertainties and made decisions informed by the input from their treatment team. This was reflected in one quote about trusting their physician’s decision about pre-treatment chemotherapy for their child:

*[Provider 2] even picked the most aggressive treatment chemotherapy for a four month old… But we were like, [Provider 2], this is the most aggressive treatment you’re going to pick. If this is the course you’re going to pick, we’re going to do whatever you say. And so, we went with it.*

#### Affective coping

Trusting the treatment team with decisions related to their child’s treatment also helped parents cope with uncertainties surrounding treatment options. Parents reported having trustworthy doctors who helped them to manage their emotions, cope with the stress of being in the hospital, and make decisions about SCID treatment. This is depicted in one parent’s description of trusting their medical team with their child’s plan for treatment and feeling calm and supported by them:

*We just had to have faith in his doctors and ask a lot of questions. They were the ones that always calmed us down and told us, like, ‘this is our plan. He's going to be okay. We really think that this is going to work.’ We really just had to listen to them*

### Coping in the treatment stage

Generally, the treatment period in the SCID journey was brief, and parents’ uncertainties at this stage focused on their child’s prognosis after treatment. Parents often reported feeling anxiety and worry about the uncertainties of their child’s treatment procedure. They often expressed fears that their child’s immune function would not improve and that the treatment would fail. Parents most frequently reported using behavioral coping to manage uncertainties that they experienced during or immediately after their child’s treatment.

#### Behavioral coping

Similar to the pre-treatment stage, parents reported coping with medical uncertainties by *playing an active role* in their child’s care. Parents often reported tracking what medications their child was given and ensuring that the nursing staff at the hospital followed hygienic protocols. Parents who had educational backgrounds in medicine reported making minor treatment suggestions which allowed them to feel in “control” at the hospital. This is reflected by one parent who said:

*When the children get the transplant, they give them a shot to help the neutrophils go up. It’s an injection and it [the child’s neutrophil count] wasn’t going up. It was going through intravenously. And I said, you know, I talked to the doctors, because they know I communicate with them like I’m part of the team. I said, “Why don’t we just give it to him subcutaneously? I think it’s going to work. Cause when he was at [Name of Another Hospital] that seemed to help him with his neutrophils.’ And they put that in him subcutaneously instead of intravenously and they got my idea. His neutrophils went up*.

### Coping in the post-treatment stage

During the period following their child’s treatment, parents experienced uncertainties related to their child’s prognosis. At this stage of the journey, one or both parents often stayed at the hospital with their child for many months, and they expressed worry about not meeting the needs of other children who remained at home. Parents who had to take extended time off from their jobs also experienced uncertainties related to medical insurance and financial resources. In the post-treatment stage, parents reported feeling anxious as they waited to hear about the outcome of their child’s treatment, but many also expressed their excitement about returning home with their child. Parents frequently reported using affective coping to manage these uncertainties.

#### Affective coping

In the post-treatment stage, parents demonstrated affective coping by *relying on practical help from family*, friends, social workers, and care coordinators to manage some of the uncertainties they experienced. Family members, for example, often took care of other children at home while the parents were in the hospital. This help comforted parents and reduced their worries about their children who remained at home. One parent depicted family support as follows:

*Well, we have a really great family support system. I’m really close with my mom and my sister and my brother. And so they were very, very supportive… Once [the child] got admitted, I never left her for a minute. Like I never left her, I did not come home. I did not leave her side. My husband went back and forth home to check on the [other children], but I never went back home. So, my mother and my mother-in-law took turns, you know, school and laundry and meals and keeping, you know, the house in order, um, taking care of my husband while he was here. Um, but we have a great family support system. So grateful for that.*

Relying on different types of assistance from others in their journey helped parents to feel supported and less overwhelmed, and better equipped to manage the uncertainties in the post-treatment stage. This practical help is reflected in one parent’s description of a social worker assisting in ways that made the parent feel less overwhelmed:

*We have always shared one vehicle. So, when we were in the hospital, my husband would be driving back and forth to work and our home and the hospital. Our social worker at [Hospital Name] was able to do so much for us. She got us parking vouchers, she was able to get us gas gift cards from—I forget what the charity was called—but we were able to get a lot of grants and help through different organizations. And she really helped us. She just did it without us really having to do much, which was great because we were so overwhelmed.*

### Coping in the new normal stage

After returning home with their child, parents experienced uncertainties related to their child’s future health and well-being and about SCID’s impact on their child’s quality of life. Parents were often worried about possible long-term effects of pre-treatment chemotherapy, for example whether it would affect their child’s ability to have children as an adult. Other uncertainties revolved around SCID’s impact on the child’s life expectancy and on relationships in the family. As the parents tried to adapt to their new life at home, they often felt overwhelmed as they grappled with ensuring their child’s safety and balancing other household responsibilities. In the new normal stage, parents most frequently used behavioral and cognitive coping to manage these uncertainties.

#### Behavioral coping

*Identifying the actionable elements of their child’s health and well-being* helped parents to cope with uncertainties. Parents often established house rules and hygiene protocols to ensure their child’s safety. Parents reported taking specific actions to improve the child’s health such as doing physical therapy to improve the child’s motor functions. One parent described focusing on her child’s physical well-being after they returned home from treatment:

*Um, you know, it’s been kind of a balancing act, and our main focus has been his physical health because we figure we can work on almost anything else later. And now we are also going to get virtual occupational therapy starting tomorrow to help us help him with some more skills that he had in school in person [transitioned to virtual care due to COVID].*

#### Cognitive coping

*Focusing on the actionable elements of their child’s health and well-being* also involved parents actively changing how they think about residual uncertainties related to their child’s health. Parents often reported attempting to think about the positive aspects of their child’s life and health. One example shared by parents was trying to focus in on their child’s current concerns. Parents described focusing on making sure that the child’s recovery is successful. This approach helped parents to remain cognizant of the progress of their child’s recovery as it unfolded, which helped them manage uncertainties about their child’s future. For example, one parent described:

*There is a chance of the transplant not being as effective. And so he’d have to get infusions like once a month. That would be the only thing [that the parent is uncertain about]. I don’t know. We haven’t really, honestly, talked about it or thought about the future. It’s more like we’re focusing on how well he’s doing and keeping him on this path. And, you know, if something like that pops up in the future, we’ll deal with it.*

### Uncertainties and ineffective coping across the SCID journey

Not surprisingly given the rare and life-threatening nature of SCID, parents described struggling to cope with their child’s diagnosis and the many uncertainties that SCID entails. Some parents reflected that, at times, especially early in the journey, they were not really managing uncertainties but just trying to survive. Parents reported difficulty resolving ongoing negative emotions and cognitions that resulted from SCID-related uncertainties, including guilt. Throughout the journey, including after the child’s return home, parents also described taking actions to manage SCID-related uncertainties that helped them in the short term but which they often ultimately recognized as detrimental to their general well-being.

#### Struggling with denial and guilt

As parents awaited a diagnosis for their child, some reported experiencing shock and a sense of denial. It was extremely difficult for them to accept that their child, who did not have any physical signs of illness, may have a life-threatening condition. Especially in the early stages of the SCID journey but sometimes for longer, some parents expressed feeling guilty about their child’s diagnosis and its lifelong uncertainties. Since SCID is a genetic condition, these parents reported a sense of responsibility for their child’s illness. One parent described her family’s struggle with guilt after their child’s SCID diagnosis:

*My husband and I really felt so much guilt. Because we brought another child into this world who could potentially have lifelong medical problems and she might not even make it.*

#### Difficulty practicing self-care and emotion regulation

During the pre-treatment and treatment stages of the SCID journey, parents had to make many decisions about their child’s care. Most of their attention was focused on the health of their child, and they often described unintentionally neglecting their own well-being. One mother described struggling to take care of her health:

*I remember going back, I was supposed to get my six -week checkup… after you have a baby, you have your checkup. And I had completely forgot about all of that. And I remember when we were in the hospital, I told the social worker like, ‘Hey, I haven’t had my checkup.’ Like I completely forgot about myself.*

As parents awaited their child’s treatment, they often described having trouble dealing with their feelings, which revolved around uncertainties pertaining to their child’s survival. One father spoke of his difficulties in processing his feelings combined with the exhaustion from excessive amounts of work:

*We went into this mode after we found out [the SCID diagnosis], where it was just, all right, let’s lock in and just do whatever we have to do…And I took that to the extreme and didn’t talk about my feelings or discuss it with anybody, where [wife] was joining these Facebook groups with these other parents. So, she was able to talk about it with other people. And I was just like, so ingrained in my work and didn’t think about it, that when I, you know, got so burned out from work, and I actually took time to sit down and think about it. It was just so overwhelming.*

#### Challenges with adjusting to life after treatment

As parents tried to return to their lives in their homes after their child’s treatment many were uncertain about their lifestyle post-treatment. Some reported struggling to adjust to their new lives and accept how drastically their lives had to change because of SCID:

*I wanted so badly for it to pretend like it was all going to be going back to the way it was before, but it was a very slow process. We started very small with like, you know, we would meet one family outside at a park and play, and then I had one family over to my house to play, and then I would clean everything right after, and then we had a few more people over to play. And then it was like very slow, I had to keep testing out different scenarios. And once I felt like he didn’t get sick from that, then we can move on. So, it was just a very slow rate integration back into the wild, if you will*.

### Uncertainties and adaptive coping across the SCID journey

As parents described coping throughout their child’s SCID journey, it became evident that over time some had adapted to ongoing SCID-related uncertainties. A few parents described continuing to use effective coping strategies to enhance their well-being. These cognitive coping strategies did not appear frequently throughout the journey but were linked to the process of adaptation as described by some parents. Parents who had learned coping strategies through previous experiences and life events, such as a death in their family or going through a cancer diagnosis, were able to apply the same strategies to their SCID journey. One parent described how past experiences helped him and his partner to intuitively rely on each other and address SCID-related stress:

*We had been through a number of hard times previously with death of her parents and things like that… And so we kind of already had a good bond and ability to cope with stressful life situations. So, even though those things were terrible, and in a way, it helped set us up to be able to deal with [child’s SCID diagnosis]*.

Other parents learned to adapt to uncertainties through their experiences with SCID. Some parents described deliberately using self-reflection and self-awareness as strategies that helped them to adapt. Both strategies helped parents to become aware of stressful triggers and to learn lessons from other strategies that proved ineffective. Parents also described how supportive relationships with their partner or community members (e.g., faith communities or other SCID parents) helped them adapt to the uncertainties of the SCID journey over time. Partner communication often helped parents to process their uncertainties, while religious groups emphasized the power of faith and community support. One parent described how a member of her faith community advised a mantra of “stop, feel, know,” which reflected encouragement to acknowledge her feelings and know that God and many people are there supporting her through her SCID journey. This parent reported deliberately utilizing this strategy even after her child’s treatment and sharing it with other SCID families. All these relationships assisted parents with processing a complex spectrum of emotions and with finding some meaning and hope in their experiences throughout the SCID journey.

Attempting to deliberately *adopt a positive mentality about their SCID journey* was another long-term coping strategy that parents utilized. For example, focusing on positive emotions and actionable elements of their journey were some coping strategies parents reported using. One parent described how she started to utilize positive thinking early in her child’s SCID journey and has kept that practice well into the new normal stage:

*That’s why I try to think positive because when I was so negative, just nothing was working out for us. Nothing’s going great. Finances sucked, like our living situation wasn’t great. Nothing was good when I was negative. So, I just decided to be positive, and I would wake up and just start our day, even though we were in isolation, and wake up, put on some music, me and [Child 1] would dance to Taylor Swift. Like we would just do anything to have a good day. And just slowly, my mind just became less cloudy and started thinking more clearly. And that’s when I think I became very self-aware of my feelings and how I react to things and how I decide to go with whatever’s thrown my way. Just try to be positive about it. But I do have my moments where I get sad or upset about things where I kind of go back to a little depression state, but I am quickly aware of it and just like, ‘Nope, not gonna let myself get back to that place.’*

## Discussion

As parents navigated through the many uncertainties they experienced over the course of their SCID journey, their coping strategies evolved. In the beginning of the journey, when parents experienced the initial shock of learning about their child’s SCID and were faced with treatment decisions, they often turned to information seeking about SCID, trusting the medical team, and taking active roles in treatment decisions. These coping efforts resonate with literature showing that individuals affected by illness, including parents whose child has been diagnosed with a life-threatening condition, often try to regain control of their situation by learning what they can about the condition and its treatments [22, 23]. In the later stages of the SCID journey, notably after parents returned home with their child, their coping strategies focused on concerns about their child’s long-term health, recovery, and quality of life. Parents often coped with these uncertainties by modifying the way they thought about their child’s illness, for example by choosing to “stay in the moment” and focus on the positive aspects of their child’s treatment and recovery. Another finding from this study is that one coping mechanism could reflect a combination of behavioral, cognitive, or affective approaches. Parents were able to be creative in being able to switch among various coping strategies when uncertainties became more threatening. At times, it was difficult to distinguish which type of coping was being described. For example, when parents reported coping by placing trust in the medical team, it was described both as the behavior or action of following the medical team’s advice and as an emotion of feeling supported by their medical team. Such emotional support allowed parents to form trusting relationships with the medical team, and in a way, to allow the treatment team to lead the process of treatment decision making. Therefore, although we presented examples as being representative of a specific category of coping (behavioral, cognitive, and affective), we acknowledge that categorizations can overlap.

Our results show that strategies in the later stages of the journey may become adaptive and implemented in an ongoing manner. Some of these adaptive coping strategies reported by parents of children with SCID emerged from discussions and interactions between partners, while others were fostered by faith and faith communities or professional mental health counseling. Previous life experiences also contributed to some parents’ ability to adapt. These findings echo the idea that adaptation is a multidimensional and dynamic process which often entails learning from experiences and previous struggles [24]. Adaptation occurs over time when stressors such as uncertainties are managed effectively and coping strategies are strategic. Within the field of chronic conditions, adaptation may be described as learning to integrate a chronic condition into one’s life while identifying ways that one’s life has been positively affected. A well-known example of positive gains from illness was reported by Dr. Shelly Taylor as “positive illusions.” She found that cancer patients often reported unexpected positive aspects of their lives that emerged from living with the threat of the uncertainties of a cancer diagnosis [25]. Over time, our participants reported that they gained more confidence in their own ability to manage stress and uncertainties. Other parents emphasized that through their SCID journey, they were able to develop new and lasting relationships within the SCID community.

Although parents often emphasized the use of constructive coping strategies, they also revealed that they had to learn effective ways of coping with SCID-related uncertainties when their approaches failed. Challenges in identifying effective coping strategies are not specific to SCID. Studies of parents of children with other rare genetic diseases also demonstrate challenges in coping with the stressors and emotional turmoil derived from their child’s condition [26]. Evidence from research into appraisal and coping has consistently found that the “match” between the source of the stressor and the coping strategy engaged is critical to its effectiveness at reducing stress and facilitating adaptation over time [8]. When there is a misalignment between stressors and coping strategies, addressing such stressors as SCID uncertainties can be very difficult. Given that SCID is a life-threatening disease, coping with the many stresses and the wide range of emotions that such a diagnosis brings can be overwhelming. At times, coping strategies that were used by parents were effective in the short term, but later proved detrimental to their general well-being. Over time, through self-awareness and introspection, parents noted that some of their coping strategies were unsustainable, resulting in parents making deliberate efforts to relearn how to effectively manage their uncertainties. Such thoughtful and adaptive processes to addressing uncertainties demonstrate that coping can be accessed both intuitively and deliberately. Although some parents described intuitively putting trust in their medical team, others described deliberately attempting to change their thought processes and behaviors.

### Study implications

Given this variability in how parents changed their coping strategies, genetic counselors and medical providers with expertise in SCID may be well positioned to help parents recognize when a coping approach is not sustainable or otherwise ineffective. They also can help explore other strategies with parents that may better address the source of the stress resulting from the uncertainties. Further, it may help parents to recognize that they have the resources to intuit an approach to coping with uncertainties that feels right to them. Providers can reinforce both approaches to help parents realize their assets for coping effectively.

Our findings suggest that new parents of a child with SCID may feel less isolated or alone in their fears about the uncertainties of their child’s condition by having contact with other parents of a child with SCID. Over time, they may find deliberate approaches to managing uncertainties. For example, new SCID parents may consider joining support or faith groups, talking with other SCID parents, keeping a journal, or confiding their fears to their partners. Although each SCID parent’s journey is unique, it shares elements with other parental experiences in ways that may help to reduce some of the stressors from uncertainties. The gains parents made in managing uncertainties over time may provide new SCID parents with hope that they too will come to manage their SCID-related challenges. The examples parents shared in this study about the successful ways they managed stressful uncertainties may help others find hope in their innate ability to accept and overcome uncertainties. When faced with critical situations and challenging decisions, parents were surprised by their own abilities to manage and navigate the complexities of the SCID journey. This sentiment is reflected in the following words of one mother: “I learned a strength I never knew I had.”

### Limitations

Although this is a first assessment of coping with uncertainties among parents of children with SCID or SCID-like conditions, the findings presented here do have limitations. First, our study participants represented a convenience sample recruited through our partner patient advocacy organization. Therefore, our findings may not be generalizable to all parents who have a child with SCID or SCID-like conditions, both nationally and internationally. Our sample also consisted predominantly of mothers. Therefore, we may have not captured all coping experiences of fathers. Second, qualitative interviews often focus on a small homogeneous group of people. Therefore, our results may not reflect the experiences of more diverse communities, including parents from other countries. Third, the categorization of coping mechanisms into behavioral, affective, and cognitive approaches can be fluid and overlapping despite our coding to represent distinction between them. We categorized these approaches to account for their most frequent use by our participants. Notwithstanding these limitations, our findings illustrate useful examples of the many uncertainties faced by SCID parents and ways that they manage the stress of those uncertainties. Identifying the ways that coping matched the sources of the stressors illustrates potentially effective ways that parents can use to manage uncertainties that can be tested as future hypotheses.

## Conclusion

Our study revealed that parents of children diagnosed with SCID use a variety of strategies to cope with SCID-related uncertainties. Parents often relied on strategies that matched their concerns and available resources for coping. These strategies were composed of behavioral, cognitive, and affective approaches. At the beginning of their SCID journey, parents often coped intuitively, which later transformed into more deliberate coping. In some instances, coping strategies used by some parents throughout their SCID journey became adaptive. Despite some challenges in coping with SCID-related stressors, parents usually reported finding ways to overcome SCID uncertainties. Findings from this study can serve as a guide for SCID-related coping both for parents whose child is newly diagnosed with SCID and for providers such as social workers, genetic counselors, and psychologists.

## Supplementary Information

**Additional file 1.** Interview Guide.

**Additional file 2.** Coping Codebook.

## Data Availability

Data supporting the conclusions in this article are included within the article itself. Further data are available from the corresponding author on request.

## Abbreviations

BMT: Bone marrow transplantation
HSCT: Hematopoietic stem cell transplantation
IRR: Interrater reliability
SCID: Severe combined immunodeficiency
