# Supplementary material for: Parental coping with uncertainties along the severe combined immunodeficiency journey
Source: Orphanet J Rare Dis. 2022 Oct 27;17:390. doi: 10.1186/s13023-022-02554-9 (PMC9615184; doi:10.1186/s13023-022-02554-9)
Supplement: Supplementary file 1 — Additional file 1. Interview Guide. [file 13023_2022_2554_MOESM1_ESM.docx]

Interview Guide

## Overview of their Story

To start our interview, it would help me to hear about your family and what you’ve been through since your child was diagnosed with SCID or a SCID-like condition. So for the first few minutes of our conversation, maybe you can share an overview of your journey so far. Then we’ll get into my questions about the uncertainties you’ve experienced along the way.

IF PARENT DOES NOT MENTION, PROBE ABOUT:

- WHAT TREATMENT(S) CHILD/CHILDREN WITH SCID HAS RECEIVED
- WHERE CHILD/CHILDREN WITH SCID IS ON THE SCID JOURNEY

## Exploring uncertainties

[DEPENDING ON WHAT THE PARENT SHARES DURING THEIR STORY] From what you shared, it sounds like [thing they shared] has been a big source of uncertainty for you. [OR]

To start, when you hear the term “uncertainties,” what comes to mind related to your family’s journey having a child with SCID?

FOR SOURCES OF UNCERTAINTY THAT THE PARENT NAMES, PROBE AS NEEDED:

- To what extent is this still a source of uncertainty for you?
  - How long was this a source of uncertainty? (Days/Weeks/Months/Years)
- What, if anything, [has helped/is helping] you manage [OR cope with] this [source of uncertainty]?
  - When there is no answer to an uncertainty, how do you manage living with it?
  - What has worked for you and for your family (or partner)?
- What (else), if anything, [would have been/would be] helpful for managing [source of uncertainty]?
- If you could design a resource for SCID parents about [source of uncertainty they are talking about] what would this look like?
  - What type of media (virtual, pamphlet, video, newsletter) would this resource be?
- If you could give advice to another SCID parent about managing [source of uncertainty they are talking about], what would you say?

IF THE PARENT NAMES MOSTLY CURRENT SOURCES OF UNCERTAINTY, PROBE ABOUT PAST SOURCES. IF THE PARENT NAMES MOSTLY PAST SOURCES, PROBE ABOUT CURRENT SOURCES. OPTIONAL WAYS TO FRAME PROBING:

- POINTS ON THE JOURNEY MAP (DIAGNOSIS, PRE-TREATMENT, TREATMENT, POST-TREATMENT, THE NEW NORMAL)
  - I’d like to hear not only about your uncertainties [now/in the past], but also uncertainties you may [have experienced in the past/be experiencing now]. For example, can you tell me about any uncertainties you experienced after your bringing [Child’s name] home from the hospital after [his/her] treatment?
- DOMAINS OF UNCERTAINTY: (EXISTENTIAL/OVERARCHING, MEDICAL, PERSONAL, PRACTICAL)
  - You’ve told me about some of your [domain of uncertainty, e.g., “Medical”] uncertainties. If you’re willing to share, I’m also interested in hearing about any [other domain of uncertainty, e.g., “Personal” or “Practical”] uncertainties you may have experienced. For example, did you experience any uncertainties about your relationships with friends or family members?

FOR [PAST/CURRENT] SOURCES OF UNCERTAINTY OR DIFFERENT DOMAINS OF UNCERTAINTY THAT THE PARENT NAMES, PROBE AS NEEDED USING THE SAME SUGGESTED PROBES ABOVE.

PRIOR TO MOVING ON TO THE NEXT SEGMENT OF THE INTERVIEW, IF PARENTS HAVE NOT YET BROUGHT UP THESE TOPICS:

- What about the point when you were choosing a treatment strategy for [Child’s name]? Can you tell me about any uncertainties you felt surrounding that choice?
- Can you tell me about any uncertainties you’ve been experiencing related to the Covid-19 pandemic and how you have been managing [OR coping with] those uncertainties?
- Across your journey with your child’s SCID, how have your uncertainties been affected by talking to other parents or hearing about their experiences with SCID?
  - Have any of your uncertainties arisen from hearing about other parents’ experiences with SCID?

## Exploring uncertainty management

Have you noticed any differences in how you respond to [OR cope with] uncertainties in your journey with [Child’s name] now compared to when [he/she] was first diagnosed?

- Tell me more about how your response has changed.
- What brought about the change?
- Do you feel that the ways you are managing uncertainty today are working better?
  - [IF YES] How so?
  - [IF NO, OR IF PARENT IS NOT VERY FAR INTO THE SCID JOURNEY] Can you imagine a day in the future when you’ll be able to better manage your uncertainties? What will it take [OR what do you need] to get there?
