# Supplementary material for: Parental coping with uncertainties along the severe combined immunodeficiency journey
Source: Orphanet J Rare Dis. 2022 Oct 27;17:390. doi: 10.1186/s13023-022-02554-9 (PMC9615184; doi:10.1186/s13023-022-02554-9)
Supplement: Supplementary file 2 — Additional file 2. Coping Codebook. [file 13023_2022_2554_MOESM2_ESM.docx]

**Coping Codes**

- 1. **Code:** Active seeking of SCID-related information (e.g., Talking to other SCID families, searching information online)
  2. **Code:** Limiting amount of information sought or attended to (e.g., trying not to look for SCID related information, looking only at sources suggested by doctors)
  3. **Code:** Communicating with others about uncertainties ***[NOT partner/spouse]***
  4. **Code:** Accepting help from others (e.g., Family helping with practical things; Medical team or social workers connecting them with resources)
  5. **Code:** Pursuit of opportunities to have important conversations with one’s partner
  6. **Code:** Playing an active role in treatment decisions (e.g., Actively talking to doctors about treatment options, making treatment related decisions, reaching out for secondary medical opinions)
  7. **Code:** Planning ahead (e.g., Packing for upcoming medical visits ahead of time, seeking information to prepare for doctor’s visit)
  8. **Code:** Active self-care (e.g., Carving out time to be alone; Engaging in important aspects of life outside SCID/parenthood; Actions to take care of one’s emotional needs)
  9. **Code:** Mimicking what others model (e.g., doing something that was suggested by other people to address SCID uncertainties)
  10. **Code:** Giving back to the community or to others (e.g., Helping other families who had a child with SCID)
  11. **Code:** Seeking help from a mental health professional (e.g., Clinical counseling)
  12. **Code:** Self-reflection: Journaling
  13. **Code:** Engaging in religious or spiritual activities (e.g., Meditation, praying)
  14. **Code:** Distracting oneself from the uncertainties
  15. **Code**: Focusing on what is actionable/controllable
  16. **Code:** Ability to talk with and lean on partner (e.g., cognitive support of knowing the partner is there/available and understands; “leaning on” includes trusting and benefiting from the spouse’s coping strategies or relationship dynamics more broadly)
  17. **Code:** Differentiating what is and is not certain
  18. **Code:** Finding ways to tolerate the uncertainties
  19. **Code:** Self-reflection (e.g., Reviewing what is and isn’t working)
  20. **Code:** Finding hope
  21. **Code:** Placing trust in the medical team-believing they are doing their best to help child (potentially expand to doctors being kind, helpful, bed-side manners)
  22. **Code:** Living one day at a time/Not dwelling on the future
  23. **Code:** Focusing on the positive (e.g., thinking about the positive aspects of the child health or recovery)
  24. **Code:** Adopting a pragmatic/realistic mentality about uncertainty (“It is what it is”)
      - Looking at “both sides of the coin,” making realistic conclusions
  25. **Code:** Relying on faith in a higher power (e.g., displaying emotional trust/faith in a higher power)
  26. **Code:** Stop feeling sorry for myself
  27. **Code:** Distracting oneself from the uncertainties (e.g., trying not to think about uncertainties)
  28. **Code:** Pretending to be okay for the sake of others
  29. **Code:** Not taking care of personal needs/health (e.g., Missing personal check-up appointments, no eating well, not sleeping)
  30. **Code:** Assigning blame to self (e.g., Expressing thoughts or feelings of guilt relate to the child’s diagnosis)
  31. **Code:** Not managing uncertainties, just surviving
  32. **Code:** Suppressing or minimizing uncertainty (negative context with a specific action, e.g., working too much)
  33. **Code:** Fixating on helping the child (negative context, e.g., at the expense of self-care or other relationships)
